# Low muscle strength and self-reported fatigue in patients on hemodialysis: findings from the SARC-HD study

**DOI:** 10.3389/fnut.2025.1583976

**Published:** 2025-05-22

**Authors:** Marvery P. Duarte, Otávio T. Nóbrega, Maryanne Z. C. Silva, Dario R. Mondini, Bruna M. Sant'Helena, Daiana C. Bundchen, Maristela Bohlke, Angélica N. Adamoli, Ricardo M. Lima, Antônio Inda-Filho, João L. Viana, Barbara P. Vogt, Maycon M. Reboredo, Heitor S. Ribeiro, the SARC-HD Study Group, Fábio A. Vieira, Priscila M. Varela, Jacqueline S. Monteiro, Marina S. Pereira, Ana C. Bainha, Emanuele P. Gravina, Abner R. Castro, Fabiana L. Costa, Luiz R. Medina, Flávio I. Nishimaru, Maria G. Rosa, Ana C. Picinato, Marco C. Uchida, Karine Pires Costa, Beatriz R. Viana, Antônia S. Almeida, Ana C. Pires, Catiussa Colling, Aparecido P. Ferreira

**Affiliations:** ^1^Faculty of Health Sciences, University of Brasilia, Brasília, Brazil; ^2^Internal Medicine Department, Botucatu Medical School, São Paulo State University, UNESP, Botucatu, Brazil; ^3^Laboratory of Applied Kinesiology, Faculty of Physical Education, Universidade Estadual de Campinas, Campinas, Brazil; ^4^IELUSC Faculty, Joinville, Brazil; ^5^Department of Health Sciences, Federal University of Santa Catarina, Araranguá, Brazil; ^6^Postgraduate Program in Health and Behavior, Catholic University of Pelotas, Pelotas, Brazil; ^7^Hospital de Clínicas de Porto Alegre, Porto Alegre, Brazil; ^8^Faculty of Physical Education, University of Brasilia, Brasília, Brazil; ^9^Research Center in Sports Sciences, Health Sciences and Human Development, University of Maia, Maia, Portugal; ^10^Graduate Program in Health Sciences, Medicine Faculty, Federal University of Uberlandia, Uberlândia, Brazil; ^11^School of Medicine, Federal University of Juiz de Fora, Juiz de Fora, Brazil

**Keywords:** dialysis, fatigue, sarcopenia, muscle weakness, tiredness

## Abstract

**Background:**

Whether low muscle strength contributes to fatigue remains poorly understood. We investigated the association between dynapenia and self-reported fatigue in patients on hemodialysis.

**Methods:**

A cross-sectional analysis of the multicenter SARC-HD study in 19 dialysis units across Brazil. Muscle strength was evaluated by handgrip strength (HGS) and five times sit-to-stand (STS-5). Low muscle strength (i.e., dynapenia) was defined based on the revised EWGSOP. Patients were stratified into four dynapenia phenotypes (i) no dynapenia; (ii) low HGS; (iii) low STS-5; and (iv) severe dynapenia (low HGS and STS-5). From the validated 36-item short-form health survey (SF-36) question about tiredness, patients self-reported their frequency of fatigue as (i) *Never or rarely*; (ii) *Sometimes*; and (iii) *Always or constantly*.

**Results:**

Among 841 patients (58 ± 15 years, 38% female, and 49% Black), the prevalences of dynapenia by low HGS, low STS-5, and severe dynapenia were 13.9, 18.8, and 12.1%, respectively. Frequency of fatigue, self-reported as “*Never or rarely*,” “*Sometimes*” or “*Always or constantly*” was 39.5, 30.3, and 30.2%, respectively. The frequency of “Always or constantly” feeling fatigued was 24.2% among patients without dynapenia, 36.5% in dynapenia by low HGS, 37.2% in dynapenia by low STS-5, and 37.8% in severe dynapenia (*p* < 0.001). Adjusted logistic regressions showed a significant association between all dynapenia phenotypes and high frequency of fatigue compared to those without dynapenia (low HGS: odds ratio [OR] = 1.91; 95% confidence intervals [CI]: 1.12–3.23; low STS-5: OR = 2.35; 95%CI: 1.50–3.69; severe dynapenia: OR = 2.73; 95%CI: 1.55–4.81).

**Conclusion:**

Patients on hemodialysis with low muscle strength were more likely to self-report a higher frequency of fatigue, independently of the dynapenia phenotype. This highlights the importance of recognizing low muscle strength as a potential contributor to fatigue in this population.

## 1 Introduction

The term dynapenia was introduced in 2008 by Clark and Manini to describe a neuromuscular disorder characterized by an age-related decline in muscle strength, meaning “*poverty of strength*” in Greek (1). Its diagnosis relies on muscle strength cutoffs based on relatively healthy and young populations. Notably, approximately half of patients with end-stage kidney disease (ESKD) on hemodialysis present dynapenia, more than twice the prevalence observed in non-dialysis counterparts (2). This is mainly explained by the accelerated aging model, a multifactorial process in part driven by the chronic inflammation associated with kidney disease progression (3). In patients with ESKD, dynapenia increases the risk of mortality by 120% (4).

Previous evidence showed that clinical- and dialysis-related variables, such as ultra-sensitive C-reactive protein (5), low serum albumin (6), vitamin D deficiency, and elevated interleukin-6 (7) are associated with muscle strength in patients on hemodialysis. Concurrently, these patients often experience burdensome symptoms, including fatigue (8–10). In this regard, fatigue is reported by up to 80% of patients on hemodialysis (11–13), and tends to worse on dialysis days (14).

Although both dynapenia and fatigue are highly prevalent and clinically impactful in patients on hemodialysis, no previous study has explored their association. From a clinical and physiological perspective, both conditions may arise from shared mechanisms, including chronic inflammation, neuromuscular impairment, and altered energy metabolism. Clarifying this association may enhance early screening and guide tailored interventions to improve physical function and tiredness. Therefore, to address this knowledge gap, our study aimed to investigate the association between dynapenia and the frequency of self-reported fatigue in patients on hemodialysis.

## 2 Materials and methods

### 2.1 Design, setting, and population

The baseline data from the SARC-HD (*SARCopenia trajectories and associations with clinical outcomes in patients on HemoDialysis*), a national multicenter cohort study, conducted at 19 dialysis units across Brazil, from October 2022 to April 2023, was used for this cross-sectional report. A more comprehensive description of the SARC-HD study is available elsewhere (15). Briefly, eligibility criteria included patients aged 18 years and older undergoing maintenance hemodialysis for at least 3 months. Exclusion criteria encompassed physical limitation that impaired assessing muscle strength and cognitive inability to respond to the self-reported question about fatigue. Written informed consent was obtained from all participants. The study was ethically approved by the institutional review board of the University Center ICESP (no. 5.418.365) and complies with the Declaration of Helsinki. Other institutional review boards also reviewed and concurred with the approval decision. The SARC-HD study is also registered at the *Registro Brasileiro de Ensaios Clínicos* (ReBEC) platform (RBR-82p87rq). All methods were performed in accordance with the relevant Brazilian and international guidelines and regulations.

### 2.2 Sociodemographic and clinical variables

Clinical and demographic data were gathered from electronic medical records by the same experienced researcher at each dialysis center. Any missing information was requested from either the patients or the medical team.

### 2.3 Assessment of muscle strength

Muscle strength was evaluated by an experienced researcher at each dialysis unit before a midweek dialysis session. A detailed description of the protocols is available elsewhere (15).

#### 2.3.1 Handgrip strength

Handgrip strength (HGS) was assessed using two hydraulic dynamometers, the Jamar (Sammons Preston Rolyan, Bolingbrook, IL, USA) or the Saehan (Saehan Corp., Changwon, Korea), depending on the availability of the dialysis unit. These two dynamometers demonstrate excellent concurrent validity, with an intraclass correlation coefficient of 0.96 (16). All assessments were conducted by the same trained evaluator at each dialysis units to ensure standardization and reduce inter-rater bias. The greatest value obtained from three repetitions was considered and reported in kilogram (kg) (17).

#### 2.3.2 Five times sit-to-stand test

Lower-limb muscle strength was evaluated using the five times sit-to-stand (STS-5) test. The shortest duration in seconds from three trials was documented (18).

### 2.4 Phenotypes and diagnoses of dynapenia

Low muscle strength (i.e., dynapenia) was defined based on the revised European Working Group on Sarcopenia in Older People (19). Low HGS was defined as <27 kg for men and <16 kg for women. Low lower-limb muscle strength was defined as >15 s to perform the STS-5 for both sexes. Patients were stratified into four phenotypes of dynapenia: (i) no dynapenia; (ii) dynapenia by low HGS only; (iii) dynapenia by low STS-5 only; and (iv) severe dynapenia (both low HGS and low STS-5).

### 2.5 Self-reported frequency of fatigue

Fatigue was self-reported by the patient and defined as *a general feeling of tiredness* (20). For this purpose, we adapted the validated 36-item short-form health survey (SF-36) question “*How much of the time during the past 4 weeks did you feel tired?*” (21). Patients were then asked, “*How often during the past weeks did you feel tired?*.” The options for answering were (i) *never or rarely*; (ii) *sometimes*; and (iii) *always or constantly*.

## 3 Statistical analysis

Continuous data are shown as mean and standard deviation unless otherwise stated, whereas categorical data as frequency and percentage. Normality of data was assessed by histogram visual inspection and by the Kolmogorov–Smirnov test. Missing data are shown in Supplementary Table 1 and no imputation has been performed. Group comparisons based on the phenotype of dynapenia were performed using the one-way ANOVA or the Kruskal-Wallis tests with Bonferroni post-hoc correction for continuous variables, while the Chi-Square or Fisher Exact tests for categorical variables. Multinomial logistic regression was conducted to investigate the association between dynapenia (*no dynapenia* group as reference) and the categories of self-reported frequency of fatigue (*never or rarely* as reference). Odds ratios (OR) and 95% confidence intervals (CI) were calculated. The adjusted model incorporated clinical and sociodemographic variables that exhibited statistical differences among the groups. The analyses were conducted using the Statistical Package for the Social Sciences (version 29.0, SPSS Inc., Chicago, USA) and GraphPad Prism (version 8.4, GraphPad Software, San Diego, USA). We considered a *p-*value < 0.05 as statistically significant.

## 4 Results

### 4.1 Characteristics of the sample

**Figure 1:**
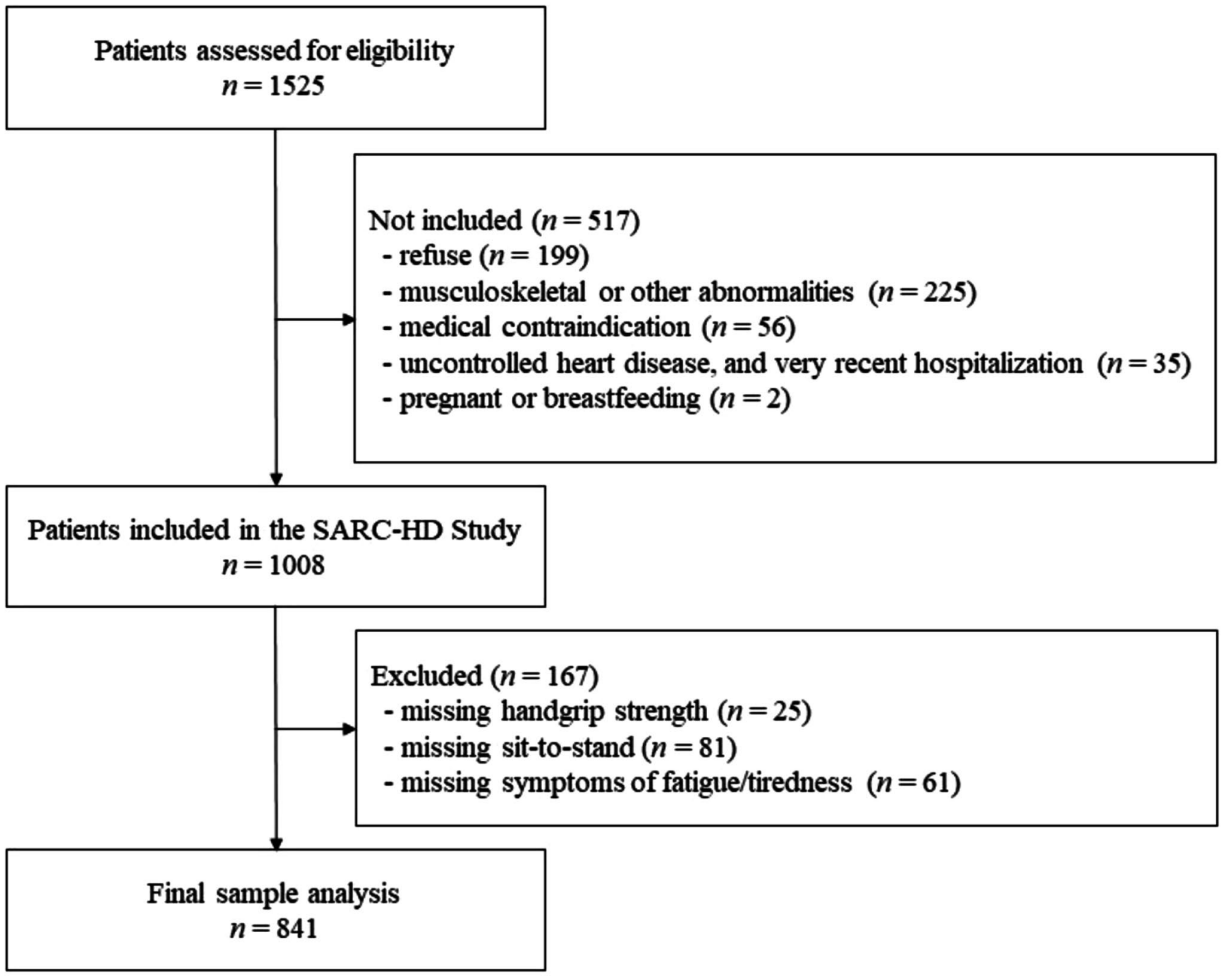
Study flowchart of patients’ recruitment.

A thousand and eight patients were included in the SARC-HD study, of whom 841 were included in the present report after exclusions for missing data (Figure 1). A total of 387 (46%) patients were classified with one of the dynapenia phenotypes. Table 1 describes the characteristics of patients according to the different phenotypes. Patients in the no dynapenia group had a lower frequency of White ethnicity (*p* < 0.001), a higher frequency of short daily dialysis (*p* = 0.038), and less diabetes (*p* < 0.001) than the dynapenia groups. Patients with severe dynapenia were older than the other groups (*p* < 0.001). There were more female patients in the dynapenia by STS group (52%; *p* < 0.001). Body mass index did not differ among groups (*p* = 0.055).

**Table 1:** Characteristics of the patients on hemodialysis accordingly to the different phenotypes of dynapenia.

	All patients	No dynapenia	Dynapenia by HGS	Dynapenia by STS	Severe dynapenia	*p*-value
*n* (%)	841	454 (54)	104 (12)	172 (21)	111 (13)	
Age (years)	57.5 ± 15.1	52.0 ± 14.0	62.7 ± 15.9^a^	62.0 ± 13.2^a^	67.9 ± 12.0^a,b,c^	<0.001
Female, *n* (%)	318 (38)	171 (38)	23 (22)	90 (52)	34 (31)	<0.001
Hemodialysis vintage, median months [IQR]	32 [13–63]	33 [16–63]	26 [10–59]	33 [15–63]	28 [10–70]	0.382
Height (cm)	166.5 ± 9.7	167.5 ± 9.4	163.7 ± 9.5^a^	166.3 ± 9.5	165.3 ± 10.5	0.002
Body weight (kg)	71.5 ± 15.8	72.7 ± 16.4	66.8 ± 13.8^a^	73.1 ± 15.4^b^	68.9 ± 14.8	<0.001
Body mass index (kg/m^2^)	25.7 ± 5.0	25.8 ± 5.1	24.9 ± 4.9	26.4 ± 4.8	25.1 ± 4.9	0.055
**Ethnicity**, *n* (%)^†^						<0.001
Black	414 (49)	239 (53)	37 (36)	94 (55)	44 (39)	
White	392 (47)	189 (42)	63 (61)	75 (43)	65 (59)	
Other	35 (4)	26 (5)	4 (3)	3 (2)	2 (2)	
**Treatment method**, *n* (%)						0.020
Hemodialysis	571 (68)	303 (67)	62 (60)	132 (77)	74 (67)	
Hemodiafiltration	270 (32)	151 (33)	42 (40)	40 (23)	37 (33)	
**Weekly frequency**, *n* (%)						0.038
Conventional (2 or 3 sessions)	583 (69)	297 (65)	73 (70)	132 (77)	81 (73)	
Short daily (≥4 sessions)	258 (31)	157 (35)	31 (30)	40 (23)	30 (20)	
**Comorbidities**, *n* (%)						
Diabetes	335 (40)	148 (33)	58 (56)	76 (44)	53 (49)	<0.001
Hypertension	703 (84)	375 (83)	87 (84)	149 (87)	92 (84)	0.686
Neuropathy	68 (8)	39 (9)	6 (6)	10 (6)	13 (12)	0.249
**Muscle strength**						
Handgrip strength (kg)	28.0 ± 10.1	32.8 ± 9.3	19.4 ± 5.0^a^	27.1 ± 7.3^a,b^	17.2 ± 5.9^a,c^	<0.001
Male	32.0 ± 10.1	38.0 ± 7.2	21.3 ± 3.8^a^	33.0 ± 5.1^a,b^	19.4 ± 5.7^a,c^	<0.001
Female	21.4 ± 6.2	24.2 ± 5.0	12.9 ± 1.9^a^	19.6 ± 6.2^a,b^	12.3 ± 2.2^a,c^	<0.001
Five times sit-to-stand (sec)	13.1 ± 5.2	10.5 ± 2.6	11.7 ± 2.2^a^	19.5 ± 5.9^a,b^	19.3 ± 4.1^a,b^	<0.001

IQR, interquartile range; HGS, handgrip strength; STS, sit-to-stand.

† Ethnicity was self-reported by the patients. Severe dynapenia is the coexistence of low HGS and low STS.

^a^, ^b^, and ^c^ indicate significant differences to the no dynapenia, dynapenia by HGS, and dynapenia by STS groups, respectively.

### 4.2 Frequency of fatigue

**Figure 2:**
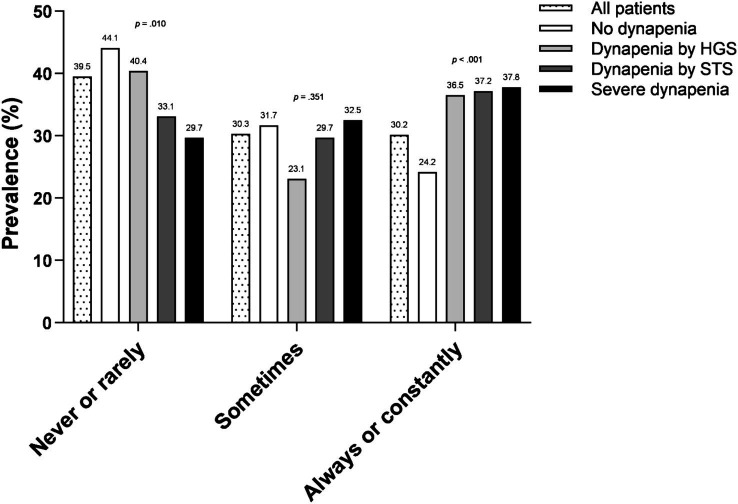
Frequency of fatigue according to the different phenotypes of dynapenia. HGS, handgrip strength; STS, sit-to-stand.

Figure 2 shows the prevalence of self-reported frequency of fatigue for the entire sample as well as stratified by dynapenia status. *Never or rarely* feeling fatigued was reported by 40% (*n* = 332) of the patients, with significantly higher proportion in the no dynapenia group (44.1%, *n* = 240; *p* = 0.010). Thirty percent (*n* = 255) of the patients reported *Sometimes* feeling fatigued, with no significant difference among groups (*p* = 0.351). *Always or constantly* feeling fatigued was found in 30% (*n* = 254) of the patients, with a significantly higher proportion in the dynapenia groups (*p* < 0.001).

### 4.3 Association of dynapenia with the frequency of fatigue

**Figure 3:**
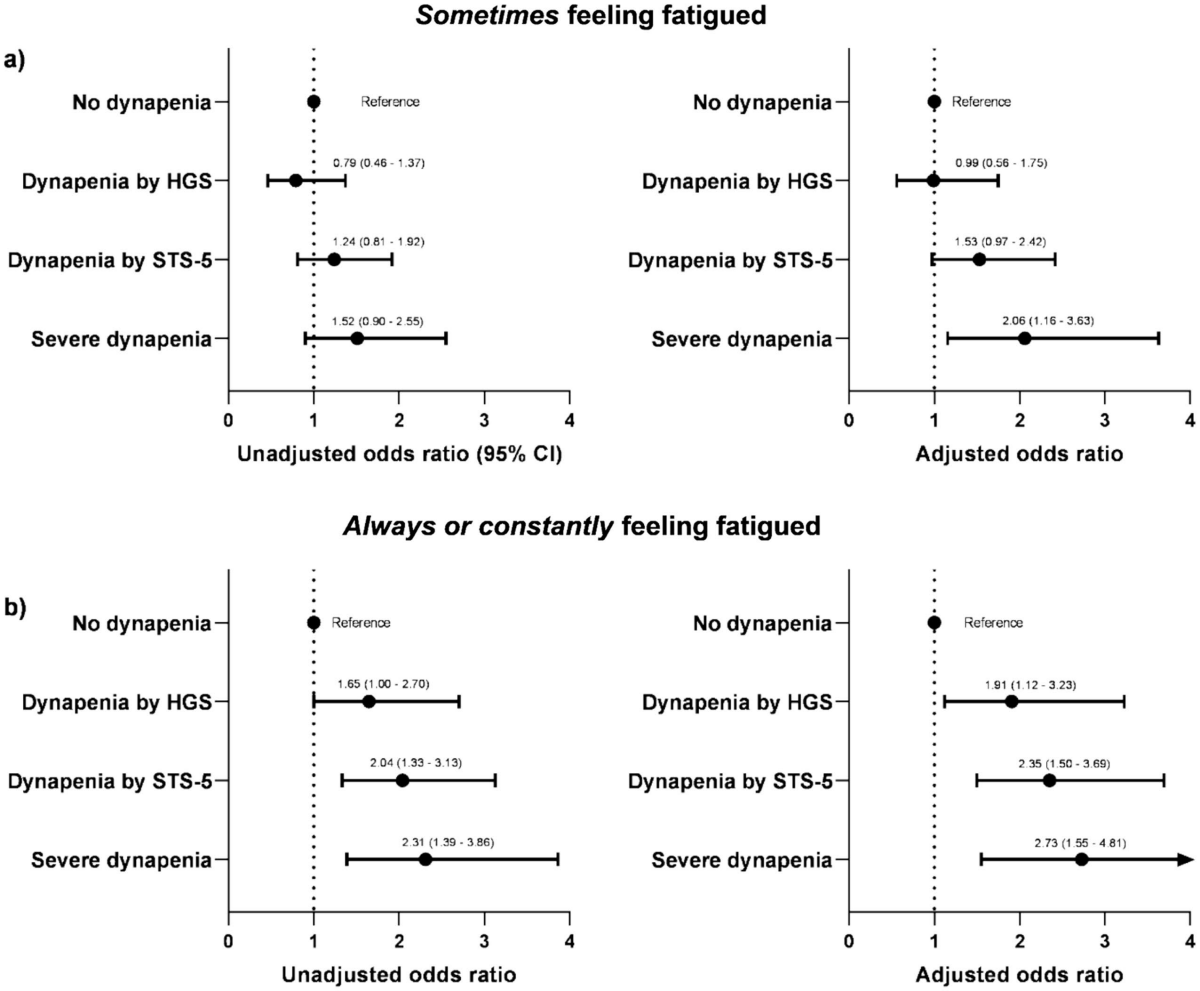
Odds ratio for symptoms of fatigue according to the different phenotypes of dynapenia. **(a)** Sometimes feeling fatigued; **(b)** always or constantly feeling fatigued. Adjusted model included age, gender, dialysis modality, treatment frequency, diabetes, body mass index, and ethnicity as covariates.

The association between dynapenia and the frequency of fatigue is shown in Figure 3. In the adjusted model for covariates (age, gender, dialysis modality, treatment frequency, diabetes, body mass index, and ethnicity), the severe dynapenia group was 106% (95% CI: 1.16–3.63) more likely to *Sometimes* feeling fatigued. Regarding the higher fatigue frequency group, all dynapenia phenotypes were independently associated with *Always or constantly* feeling fatigued (unadjusted and adjusted models). In the adjusted model, the dynapenia by HGS, dynapenia by STS, and severe dynapenia groups had a 91% (95% CI: 1.12–3.23), 135% (95% CI: 1.50–3.69), and 173% (95% CI: 1.55–4.81) higher risk for *Always or constantly* feeling fatigued, respectively.

## 5 Discussion

This cross-sectional study investigated the association between different phenotypes of low muscle strength (i.e., dynapenia) and the frequency of self-reported fatigue in patients on hemodialysis. Our findings demonstrated that, independently of the phenotype, dynapenia was associated with a higher frequency of fatigue. When referring to the *Always or constantly* feeling of fatigue, patients with dynapenia had approximately a twofold higher risk of feeling it compared to those without the phenotype. Regarding the *Never or rarely* feeling of fatigue, the dynapenia defined by low HGS group had similar rates than the no dynapenia group, whereas the groups with dynapenia by STS and severe dynapenia had a lower proportion of *Never or rarely* feeling fatigued. Taken together, these findings provide support for the concept that low muscle strength is associated with self-reported fatigue and should be considered in interventions aiming to address fatigue in patients on hemodialysis.

Fatigue has been recently recognized as one of the most important patient-reported outcome measures and a top research priority for patients living with ESKD (22), yet little is known about its epidemiology, pathogenesis, and treatment. We found that 60% of our sample reported some frequency of fatigue, with approximately half of them feeling it *Always or constantly*. Bossola et al. found that fatigue was associated with a high prevalence of physical symptoms, such as muscle soreness, bone or joint pain, and shortness of breath (11). These symptoms might influence physical activity patterns and muscle strength, making these patients more prone to develop dynapenia. Patients experiencing dynapenia typically need a higher proportion of their physical capacity to accomplish specific daily life tasks. In support of this assumption, Matsufuji et al. identified that handgrip strength was independently associated with activities of daily living, especially eating, grooming, and bathing (23). Interestingly, knee extension was less strongly associated with such activities of daily living. Thus, there seems to be a different pattern of this association regarding the limb of strength assessment. Our findings on the frequency of fatigue showed that dynapenia by STS, but not by HGS, was associated with a higher proportion of *Never or rarely* feeling fatigued. This can be explained by a more exacerbated loss of muscle mass and function in the lower limbs, which constitute a larger portion of total muscle mass and are more directly involved in mobility-related activities, such as walking, standing, and climbing stairs, activities often impaired in individuals who report a high frequency of fatigue. In older adults, overtime loss of leg lean mass was associated with declines in muscle strength, which may increase perceived effort and fatigue in those with dynapenia in the lower limbs (24). Moreover, lower limb dynapenia may reflect more pronounced neuromuscular dysfunction, involving mechanisms such as impaired neuromuscular transmission and reduced oxidative capacity, which are known contributors to fatigue (25). These alterations compromise energy production and muscular efficiency, particularly during functional activities, potentially explaining the stronger association between lower limb strength and fatigue perception in our study.

Dynapenia was associated with a higher frequency of *Always or constantly* feeling fatigued in our sample. There is little previous evidence on the topic in patients with ESKD. Molfino et al. found that patients with fatigue had lower handgrip strength than their no fatigue counterparts (18.7 ± 5.0 *vs*. 26.3 ± 10.1, respectively). This phenomenon may be explained by neuromuscular impairments due to altered skeletal muscle excitability and nerve excitability (26), confirmed by our findings where independently of the phenotype of dynapenia, a significant association was found with fatigue. In elderly patients with chronic kidney disease not on dialysis, Chatrenet et al. assessed neuromuscular fatigability through a handgrip task (27). Their findings showed that patients with chronic kidney disease had higher neuromuscular fatigability than controls, mainly explained by an early-phase contraction impairment associated with a deficiency in motor unit recruitment. This confirms that chronic kidney disease may promote neuromuscular disturbances, which might increase the feeling of tiredness when performing daily physical activities.

In this scenario, exercise interventions may be useful to prevent or delay muscle strength decline and potentially modify fatigue symptoms, as previously described in modest clinical trials (28, 29). Indeed, patients recognize exercise interventions to be an important fatigue modifiable factor (30), thus we suggest clinicians should consider prescribing resistance exercise for patients who experience symptoms of fatigue, especially in those with dynapenia.

Limitations are worth mentioning in our study. The frequency of fatigue was assessed using an adapted question from SF-36, a validated instrument not designed to effectively assess fatigue. Given that fatigue is a multidimensional symptom that involves physical, emotional, and cognitive aspects, more comprehensive tools such as the FACIT-Fatigue or the Multidimensional Fatigue Inventory would have provided a more detailed evaluation (31). Nonetheless, brief self-reports are widely used in clinical research due to their feasibility and low respondent burden (32). Moreover, as we were not able to apply a well-designed questionnaire for fatigue, this did not allow us to score the severity of fatigue, which would have been important to statistically identify the degree of association with measures of muscle strength (i.e., HGS and STS-5 performance). Moreover, we were unable to collect hemoglobin levels to diagnose anemia, a well-known risk factor for fatigue, which could modify the dynapenia-fatigue association. However, the key strengths of our study were the multicenter design with a large nationally representative sample size, which minimizes single-center bias. Also, we objectively assessed muscle strength using widely validated instruments and methods, different than previous studies that assessed self-reported physical function through scales or questionnaires.

## 6 Conclusion

In conclusion, our results showed that patients with low muscle strength, independently of the dynapenia phenotype, were more likely to have a higher frequency of self-reported fatigue compared to those without dynapenia. This emphasizes the significance of identifying low muscle strength as a potential modifiable factor for fatigue in this population. Therefore, future research may explore interventions that improve muscle strength and if such improvements may reduce the frequency and severity of fatigue.

## Data Availability

The raw data supporting the conclusions of this article will be made available by the authors without undue reservation, by contacting the corresponding author.

## Corporate Authors of SARC-HD Study Group

Fábio A. Vieira^1^, Priscila M. Varela^13^, Jacqueline S. Monteiro^13^, Marina S. Pereira^12^, Ana C. Bainha^12^, Emanuele P. Gravina^12^, Abner R. Castro^12^, Fabiana L Costa^2^, Luiz R. Medina^14^, Flávio I. Nishimaru^15^, Maria G. Rosa^16^, Ana C. Picinato^16^, Marco C. Uchida^3^, Karine Pires Costa^5^, Beatriz R. Viana^5^, Antônia S. Almeida^6^, Ana C. Pires^6^, Catiussa Colling^7^, Aparecido P. Ferreira^1^.

^13^DaVita Kidney Care Brazil, Brasilia, Brazil.

^14^PlanoAr Reabilitação, São Paulo, Brazil.

^15^NefroClass, Paulínia, Brazil.

^16^Renal Quality, Jundiaí, Brazil.
