# Supplementary material for: Low muscle strength and self-reported fatigue in patients on hemodialysis: findings from the SARC-HD study
Source: Front Nutr. 2025 May 22;12:1583976. doi: 10.3389/fnut.2025.1583976 (PMC12137102; doi:10.3389/fnut.2025.1583976)
Supplement: Supplementary file 1 [file Table_1.pdf]

Supplementary Material

Table S1. Missing data.

	**Absolute (n)**	**Relative (%)**
Age	0	0
Sex	0	0
Dialysis modality	0	0
Weekly frequency	0	0
Dialysis vintage	1	0.1
Ethnicity	1	0.1
**Comorbidities**		
Diabetes	2	0.2
Hypertension	1	0.1
Neuropathy	3	0.4
**Body composition**		
Height	1	0.1
Body weight	6	0.7
Body mass index	5	0.6
**Muscle strength**		
Handgrip strength	0	0
Five-time sit-to-stand	0	0
