# Supplementary material for: Low muscle strength and self-reported fatigue in patients on hemodialysis: findings from the SARC-HD study
Source: Front Nutr. 2025 May 22;12:1583976. doi: 10.3389/fnut.2025.1583976 (PMC12137102; doi:10.3389/fnut.2025.1583976)
Supplement: Supplementary file 2 [file Table_2.pdf]

Supplementary Material 1. Full list of the SARC-HD Study members

Centers (Federal District): 1) DaVita Advance, Brasília, Federal District
2) DaVita Águas Claras, Águas Claras, Federal District
3) DaVita Alvorada, Brasília, Federal District
4) DaVita Ceilândia, Ceilândia, Federal District
5) DaVita Gama, Gama, Federal District
6) DaVita JK, Taguatinga, Federal District
7) DaVita Asa Sul, Brasília, Federal District
8) DaVita Pacini, Brasília, Federal District
Principal Investigator: Heitor S. Ribeiro, PhD
Coordinator: Marvery P. Duarte, MSc
Collaborating researchers: Fábio A. Vieira; Jacqueline Monteiro, MD; Priscila Varela, PT; Victor Baião, MSc; Ricardo M. Lima, PhD; Antônio Inda-Filho, PhD; Aparecido Ferreira, PhD; Otávio T. Nóbrega, PhD.

Center: NefroClass, Paulínia, São Paulo
Principal Investigator: Marco C. Uchida, PhD
Coordinator: Dário R. Mondini
Collaborating researchers: Luiz Medina, MS; Luiza Sad, PT; Flávio Nishimaru, MD

Center: Renal Quality, Jundiaí, São Paulo
Principal Investigator: Marco C. Uchida, PhD
Coordinator: Dário R. Mondini
Collaborating researchers: Luiz Medina, MSc; Luiza Sad, PT; Maria Gabriela Rosa, MD

Center: Unidade de Diálise do Hospital das Clínicas da Faculdade de Medicina de Botucatu (HCFMB), Botucatu, São Paulo
Principal Investigator: Maryanne Z. C. Silva, PhD
Coordinator: Maryanne Z. C. Silva, PhD
Collaborating researchers: Fabiana L. Costa, MSc; Isabele C. Rodrigues; Paula T. Presti; Tabata M. Silva

Center: DaVita Bauru, Bauru, São Paulo
Principal Investigator: Clara Rosa, PhD
Coordinator: Henrique Disessa, MSc
Collaborating researchers: None.

Centers (Juiz de Fora): 1) DaVita Juiz de Fora, Juiz de Fora, Minas Gerais
2) DaVita Rio Branco, Juiz de Fora, Minas Gerais
Principal Investigator: Maycon M. Reboredo, PhD
Coordinator: Marina Silveira, RD
Collaborating researchers: Emanuele P. L. Gravina, Ana C. C. Bainha

Center: Clínica de Nefrologia de Araranguá, Araranguá, Santa Catarina
Principal Investigator: Daiana Bundchen, PhD

Coordinator: Daiana Bundchen, PhD
Collaborating researchers: Christine Zomer Dal Molin, Camila Rocha Vignali, Beatriz Rocha Viana, Karine Pires Costa, Juliana dos Santos Raimundo, Laura Polo, Adriane Maria Horn, Lucas Alves Pizzutti, Gabrielli Vieira Carrer, Laís C. Carvalho, Josué dos Santos Barbosa Júnior, Barbara Marjorie Schwab, Rafaela Aguiar Rosa

Center: Fundação Pró-Rim, Joinville, Santa Catarina
Principal Investigator: Bruna M. Sant'Helena, PhD
Coordinator: Bruna M. Sant'Helena, PhD
Collaborating researchers: Rodolfo Nunes Bittencourt, Maria Cecília Kohler Panno

Center: Unidade de Terapia Renal Jorge Bandarra Westphalen do Hospital São Vicente de Paulo, Cruz Alta, Rio Grande do Sul
Principal Investigator: Rodrigo Krug, PhD
Coordinator: Rodrigo Krug, PhD
Collaborating researchers: Paulo Ricardo Moreira, PhD, Thais Severo Dutra, Jailton Possebom Marsola, Anny Beatriz Somavilla, Eduarda Martins Machado, Taiene Rodrigues, Amiria Teixeira Santana

Center: Unidade de Hemodiálise do Hospital de Clínicas de Porto Alegre (HCPA), Porto Alegre, Rio Grande do Sul
Principal Investigator: Angélica Adamoli, PhD
Coordinator: Angélica Adamoli, PhD
Collaborating researchers: Catiussa Colling, Rodrigo Jacobsen, Sabrina Rodrigues da Silva, Deise dos Santos Farias, Júlia Rodrigues, Raíssa Teixeira

Center: Hospital Universitário São Francisco, Pelotas, Rio Grande do Sul
Principal Investigator: Maristela Bohlke, PhD
Coordinator: Maristela Bohlke, PhD
